# Maternal risk factors for low birthweight and macrosomia: a cross-sectional study in Northern Region, Ghana

**DOI:** 10.1186/s41043-023-00431-0

**Published:** 2023-08-29

**Authors:** Silas Adjei-Gyamfi, Bashiru Musah, Abigail Asirifi, John Hammond, Paul Armah Aryee, Sato Miho, Hirotsugu Aiga

**Affiliations:** 1 Present Address: School of Tropical Medicine and Global Health, Nagasaki University, 1-12-4, Sakamoto, Nagasaki City, 852-8523 Japan; 2 Savelugu Municipal Hospital, Ghana Health Service, P.O. Box 45, Savelugu, Northern Region Ghana; 3 Seventh Day Adventist Hospital, Christian Health Association of Ghana, P.O. Box 24, Wiamoase, Ashanti Region Ghana; 4 Central Regional Health Directorate, Ghana Health Service, P.O. Box 63, Cape Coast, Central Region Ghana; 5 Department of Nutritional Sciences, School of Allied Health Sciences, University for Development Studies, P.O. Box TL 1883, Tamale, Northern Region Ghana

**Keywords:** Abnormal birthweight, Ghana, Low birthweight, Macrosomia, Northern Region, Risk factors

## Abstract

**Background:**

Abnormal birthweights are critical public health challenges accountable for most non-communicable diseases and perinatal mortalities. Regardless of the myriad of mixed evidence on maternal factors responsible for abnormal birthweight globally, most of these findings are attained from urban and rural settings. This study serves as one of the key pieces of evidence in view of the increasing prevalence of abnormal birthweight particularly in some parts of semi-rural Ghana. The study, therefore, aims to estimate the prevalence of abnormal birthweight and identify some possible maternal risk factors for abnormal birthweight in Northern Ghana.

**Methods:**

A retrospective cross-sectional study was conducted in Savelugu municipality from February–March 2022. A total of 356 mothers aged 16–46 years, having a neonate and attending postnatal care service, were recruited as study participants. Data were collected from maternal and child health record books and through structured interviews. To identify the maternal risk factors for abnormal birthweight, chi-square/Fischer’s exact test and multinomial logistic regression were employed as bivariate and multivariate analyses, respectively, at 95% confidence level.

**Results:**

Prevalence rates of low birthweight and macrosomia were 22.2% and 8.7%, respectively. Maternal anaemia in first trimester (AOR 3.226; 95% CI 1.372–7.784) and third trimester (AOR 23.94; 95% CI 7.442–70.01) of gestation was strong predictors for low birthweight. Mothers belonging to minority ethnic groups (AOR 0.104; 95% CI 0.011–0.995); mothers who had ≥ 8 antenatal care visits (AOR 0.249; 95% CI 0.103–0.602); and mothers having neonates whose birth length > 47.5 cm (AOR 0.271; 95% CI 0.113–0.651) had reduced odds for low birthweight. Alternatively, mothers with gestational weeks ≥ 42 (AOR 23.21; 95% CI 4.603–56.19) and mothers from the richest households (highest socioeconomic homes) (AOR 14.25; 95% CI 1.638–23.91) were more likely to birth to macrosomic infants.

**Conclusion:**

The prevalence rates of low birthweight and macrosomia were relatively high. Anaemia in the first and third trimesters was strong determinants of low birthweight. Being minority ethnic group, frequency of antenatal visits, and childbirth length reduced the risk of low-weight births. Advanced gestational age and socioeconomic status of mothers were also predictors of macrosomia. Hence, nutrition counselling, community health education, and promotion of lifestyle improvement coupled with strengthening of health service delivery are recommended interventions.

## Introduction

Birthweight is the most measurable outcome of pregnancy categorized into normal, low, and high birthweights [1]. Globally, abnormal birthweight consisting of low and high (macrosomia) birthweights serves as not just the major causes of child deaths and morbidity [2] but also relevant contributors to long-term diseases such as diabetes and hypertension [3]. Other evidenced consequences of abnormal birthweight are stunting, wasting, and underweight [4]. These abnormal birthweights are significant interference to the realization of the Sustainable Development Goal 3 target of 25% reduction in child mortality by 2030 [5]. It is therefore necessary to direct interventions towards the events of pregnancy as most abnormal birthweight originates from intrauterine development [3]. Though low birthweight is a complex birth outcome indicator that includes overlap between preterm and small-for-date newborns [6], it is defined by UNICEF and WHO, as the weight at birth less than 2500 g, while macrosomia represents birthweight equal to or greater than 4000 g [7].

It is estimated that yearly 15% to 20% of all births are less than 2500 g, representing more than 20 million births worldwide. In sub-Saharan Africa, the prevalence of low birthweight is around 13.0% which is more than double-folds in the developed countries [6]. Correspondingly, the predominance of low birthweight among some African countries such as Senegal, Burkina Faso, Malawi, Ghana, and Uganda was 15.7%, 13.4%, 12.1%, 10.2%, and 10.0% in a cross-sectional study [8]. However, much attention has not been placed on macrosomia because it is misperceived as a sign of good nutrition in most developing countries. Meanwhile, macrosomic infants develop similar consequences as small-weight infants [3, 9] and so adequate devotion should be given to macrosomic infants as well. In a systematic review, the approximate prevalence rates of large-weight neonates were 7.0%, 8.0%, 9.0%, and 15.0% in Argentina, Uganda, Paraguay, and Algeria, respectively [10]. Moreover, a cohort study in China reported macrosomia cases of 7.8% among pregnant women with gestational diabetes [11], while a prevalence of 3.0% was estimated in retrospective inquiry in Southern Ghana [12].

According to Ugwa et al., maternal anthropometry including weight, height, and body mass index (BMI) is a key indicator during antenatal care (ANC) and must not be underpinned in the healthcare system [13]. Evidence has shown that there is an association between maternal body metrics and abnormal birthweights [14], although some findings proved the antagonistic way [15]. A large study among Chinese pregnant mothers discovered an association between pre-gestational overweight and macrosomia. Furthermore, inadequate gestational weight gain showed strong relationship with low birthweight [16]. A systematic review in 2017 also reiterated an association between low pre-pregnancy BMI and small-size children [17]. In South Africa and some parts of Brazil, maternal factors for low birthweight were old age, low educational level, primiparity [18], fewer ANC visits, and prematurity [19]. In Northern Ghana, female infants, multiparity, and rural residential status were associated with children born with low weight [20]. Alternatively, Nigeria Demographic and Health Survey identified male infants, overweight mothers, maternal higher education, and lesser ANC visits as significant risk determinants for  macrosomic births in semi-urban cities [21]. Multiparity was also correlated with large-birth children in Southern Ghana [12]. Worldwide, gestational diabetes mellitus (GDM) is identified as critical risk factor for macrosomia, although numerous GDM conditions are underestimated in most developing countries including Ghana [22].

Despite ongoing interventions which have been evidenced to have productive impact on pregnancy outcomes such as focused antenatal care; nutrition counselling and education; girls’ iron folic acid tablet supplementation; exclusive breastfeeding; micronutrient supplementation; and malaria prevention through the distribution of long-lasting insecticide nets, and intermittent preventive treatment in pregnancy [23, 24], abnormal birthweights are still increasingly recorded in the various health facilities in Ghana [25]. While institutional skilled deliveries have slightly increased from 98.0% (2017) to 98.3% (2019), the burden of low birthweight in Savelugu municipality also rocketed from 19.0% to 35.0% [26]. As usual, the data for macrosomia are always a matter of no concern in this semi-rural municipality. It could be necessary to explore if this high prevalence of abnormal birthweight is attributed to some maternal-related factors.

Moreover, as evidenced in most developing countries, Ghana is also encountering nutrition transition where maternal under- and over-nutrition coexist reflecting the double-burden of malnutrition situation [27]. The prevalence rates of overweight and obesity among mothers of reproductive ages are 27.4% and 18.4% in urban mothers, and 18.0% and 6.8% among rural mothers, respectively [28]. The same study revealed lower BMI in Northern Ghana as compared to Southern hemisphere of the country. Considering this paradox with the increasing risk of obesity in adult lifetime at both extremities of the birthweight spectrum, it is germane to investigate factors contributing to abnormal birthweight in Northern Ghana where there is existence of stable economic development despite the current high poverty rate in the region [23]. Additionally, limited work has been done on this subject matter among Ghanaian semi-rural population. The study could contribute to the understanding of issues related to abnormal birthweight due to the present and future burden of chronic diseases in Ghana [25]. Hence, this study aims to identify risk factors for abnormal birthweight in Northern Region, Ghana.

## Methods

### Study design

A retrospective cross-sectional study was conducted in Savelugu municipality, Northern Region, Ghana, to estimate the prevalence of low birthweight and macrosomia among children of mothers attending postnatal care services and identify maternal factors responsible for these abnormal birthweights.

### Study setting and participants

Savelugu municipality is located in northern part of Northern Region of Ghana. Dagomba is the major ethnic group and agriculture accounts for the major source of income in the municipality. The municipality has five major public health facilities with 13 operational community-based health planning and services (CHPS) zones. The total number of reproductive-aged women in the municipality was 40,533 with an annual expected number of pregnancies of 6,700 in 2019 [26]. Antenatal care (ANC) and postnatal care (PNC) coverages were 99.0% and 98.0%, respectively. Also, maternal and child health record book (MCHRB) distribution coverage and mothers’ use of the MCHRB for maternal and child health (MCH) services stood at 100% as of 2019 [26].

This study targeted nursing/lactating mothers having a neonate and living in the municipality. Mothers who possessed MCHRB and seeking first-day PNC services at the health facilities were recruited, while mothers with twin deliveries, home deliveries, and cardiovascular diseases were excluded from the study.

### Sample size and sampling methods

The sample size (*n*) was determined using Cochran’s (1977) formula; *n* =  $\frac{{(Z_{ \propto /2} )^{2} {\text{ X }}p\left( {1 - p} \right)}}{{d^{2} }}$. Prevalence rates (*p*) of low birthweight and macrosomia in urban Northern Ghana were previously reported to be 29.6% and 10.5%, respectively [20]. Using precision (*d*) of 0.05, and standard *z* score $\left( {Z_{\infty /2} } \right)$ which corresponds to 95% confidence level of 1.96, the calculated sample sizes were 320 and 144 for low birthweight and macrosomia, respectively. Larger sample size (= 320) was chosen since it satisfied the sample size for both low birthweight and macrosomia. Considering 10.0% non-response rate, 356 mothers were employed as final sample size. Applying probability proportional to size approach, sample sizes for the five major public health facilities were estimated (Table 1). A total of 356 lactating mothers were randomly selected by flipping a coin, using the daily PNC registry from the respective health facilities.

**Table 1:** Description of estimated facility sample size and sampling procedure at the study site

		Diare health centre	Moglaa health centre	Pong-Tamale health centre	Savelugu health centre	Savelugu municipal hospital	Savelugu municipality (total)
(a)	Total number of first PNC visits at targeted health facilities during 2020	2220	346	852	1198	3426	8042
(b)	Facility coverage [= (*a*)/8042]	27.6%	4.3%	10.6%	14.9%	42.6%	100%
(c)	Number of mothers to be drawn for each facility [= (*b*) × 356]	98	15	38	53	152	356
(d)	Estimated number of mothers visiting PNC per day [= (*a*)/52weeks/5 days]	8.5	1.3	3.3	4.6	13.2	30.9
(e)	Expected number of interviews per day [= (*d*)/2]	4.3	0.65	1.65	2.3	6.6	15.5
(f)	Total number of working days [= (*c*)/(*e*)]	23	23	23	23	23	23
		Team A (2 data collectors)	Team B (1 data collector)	Team C (1 data collector)	Team D (1 data collector)	Team E (2 data collectors)	

### Data collection

Pretested structured questionnaires installed onto Open Data Kit (ODK) version 2021.2.4 (Get ODK Inc., San Diego, USA) of handheld tablet devices were administered from 1 February to 31 March 2022 through seven enumerators.

Majority of data were collected from MCHRB which included: maternal anthropometry (pre-pregnancy BMI) determined from parameters measured in the first trimester of pregnancy; gestational weight gain (GWG) which was estimated as weight difference of mothers, measured within one week prior to delivery and the one recorded at the first ANC visit (first trimester) [29]; anthropometric characteristics of neonates including birthweight and birth length [30]; obstetric information on parity, gravidity, and birth order;   antenatal data on frequency of ANC visits, iron folic intake, tetanus-diphtheria immunization, intake of anthelminthic drugs, and sulphadoxine–pyrimethamine (SP) doses during pregnancy [18, 19, 23]; and clinical and nutritional information on haemoglobin levels, GDM, and malaria episodes [20, 23]. According to American Institute of Medicine, since foetal weight gain in the first trimester is low (approximately 1kg) [31], the mother’s weight at first ANC visit (first trimester) is regarded as an appropriate proxy for pre-pregnancy weight [29]. Parity is considered as the number of times a mother has given birth to neonates (foetuses) with gestational age of at least 28 weeks, regardless of whether the neonate was born dead or alive, while gravidity was regarded as number of pregnancies [20, 23].

Remaining data were collected through structured interviews with mothers in locally spoken language (Dagbani) consisting of: socio-demographic characteristics of each respondent including age, marital status, educational level, ethnic group, religion, sex of neonate, and household size [18, 19, 23]; socio-economic characteristics made up of mothers’ occupation, possession of properties, type of house, type of household fuel, and source of drinking water [32]; and maternal knowledge on BMI and birthweight on their understanding on low birthweight and macrosomia, its causes, effect, and relationship with mothers’ weight.

### Data analysis

Statistical data analyses were performed by using STATA version 17.0 (Stata Corporation, Texas, USA) with all analyses determined at 95% confidence level. The data attained were transferred from ODK platform onto Microsoft excel version 16.6. Birthweight was grouped into low birthweight (< 2500 g), normal birthweight (≥ 2500 to 3999 g), and macrosomia (≥ 4000 g), whereas pre-pregnancy BMI was categorized into underweight (< 18.5 kg/m^2^), normal (≥ 18.5 to 24.9 kg/m^2^), and overweight/obese (≥ 25.0 kg/m^2^) using 2004 WHO standard criteria. Parity was classified into primiparity (0–1 delivery) and multiparity (≥ 2 deliveries), while gravidity was grouped into primigravida (0–1 pregnancy) and multigravida (≥ 2 pregnancies) [23]. GWG was dichotomized into ≥ 6 kg and < 6 kg [33]. Childbirth length was categorized into ≤ 47.5 cm and > 47.5 cm, respectively [30]. ANC visits were classified into ≥ 8 visits and < 8 visits using 2016 WHO ANC model [34]. Categorizations made for gestational age at birth were preterm (< 37 weeks), normal-term (≥ 37–41 weeks), and post-term (≥ 42 weeks), while that for maternal haemoglobin levels in pregnancy trimesters were anaemia (< 11 g/dL), normal (≥ 11–13.1 g/dL), and polycythaemia (≥ 13.2 g/dL) [23]. Household wealth index was calculated based on household assets and housing quality and was used as proxy indicator for socioeconomic status of mothers. By using principal component analysis, the socioeconomic status of mothers was then categorized into five groups (thus, wealth quintiles: poorest, poorer, middle, richer, richest) [35]. Maternal knowledge score was calculated for each respondent. Correct responses were scored one point, while incorrect responses did not receive any point for the knowledge-related questions. A composite knowledge score was calculated using 19 items and dichotomized into two (adequate, inadequate) with a possible lowest score of zero (0) and highest score of 19 [36].

Bivariate analyses (chi-square/Fisher’s exact tests) were explored to estimate relationship between dependent variable (birthweight) and each independent (background) variable. The independent variables were made up of categorical variables, whereas the dependent variable was nominal (thus, low birthweight, normal birthweight, and macrosomia).

In multivariate analyses, multinomial logistic regression was used to build the final model to identify factors for abnormal birthweight (low birthweight and macrosomia) by applying simultaneous variable entry. During the logistic analyses, birthweight was entered as low birthweight = 1, normal birthweight = 2, and macrosomia = 3 where normal birthweight was used as the base outcome (reference point). The independent variables with significant bivariate association with birthweight (*p* < 0.05 in chi-square/Fisher’s exact test) were chosen as possible independent variables for logistic analyses. Multicollinearity was checked between the possible independent variables before employing them in the logistic analyses. Chi-square/Fisher’s exact test and variance inflation factor (VIF) were used to address multicollinearity between the variables. During chi-square testing, whenever there was significant association (*p* < 0.05) between the two categorical variables, the one possessing lesser p value with the dependent variable was chosen. Moreover, VIF was used to confirm multicollinearity between the variables. During the testing, the variable with VIF less than 10 was chosen for logistic analyses.

## Results

### Socio-demographic characteristics of respondents and prevalence of abnormal birthweight

All the data from the 356 sampled mothers (with their live children, who were all in their neonatal period) were included for analysis due to non-refusal, and they all met the inclusion and exclusion criteria. The mean age ± sd of the mothers was 27.25 ± 5.44 years with most mothers (86.5%) found between 20 and 35 years of age. Greater proportion of the mothers were married (89.6%), practiced Islamic religion (87.6%), belonged to Dagomba/Mamprusi ethnic group (76.7%), and were self-employed (50.8%). Majority of neonates (70.2%) were older than one week of age, while male neonates (56.2%) were slightly more than females (43.8%). Surprisingly, majority of male neonates (63.6%) had abnormal birthweight. The prevalence rates of low birthweight and macrosomia were 22.2% (95% CI 17.9–26.9%) and 8.7% (95% CI 4.9–12.1%), respectively (Table 2).

**Table 2:** Socio-demographic and socioeconomic characteristics of respondents and prevalence of abnormal birthweight (*N* = 356)

Background characteristics	Frequency distribution		Bivariate analysis			
	Frequency	Proportion	Low birthweight	Macrosomia	Normal birthweight	*p* value†
	*n*	%	*n* (%)	*n* (%)	*n* (%)	
*Age group of mothers*^a^						
< 20 years of age	18	5.1	7 (8.9)	1 (3.2)	10 (4.1)	0.430
20–35 years of age	308	86.5	67 (84.8)	28 (90.3)	213 (86.6)	
> 35 years of age	30	8.4	5 (6.3)	2 (6.5)	23 (9.3)	
Mean = 27.25, sd = 5.44						
*Marital status*						
Married	319	89.6	68 (86.1)	27 (87.1)	224 (91.1)	0.454
Divorced/Widowed	7	2.0	3 (3.8)	0 (0.0)	4 (1.6)	
Single	30	8.4	8 (10.1)	4 (12.9)	18 (7.3)	
*Educational level*						
No education	121	34.0	25 (31.7)	11 (35.5)	85 (34.5)	0.414
Primary school	46	12.9	15 (19.0)	4 (12.9)	27 (11.0)	
Junior high school	46	12.9	14 (17.7)	5 (16.1)	27 (11.0)	
Senior high school	105	29.5	17 (21.5)	8 (25.8)	80 (32.5)	
University/graduate school	38	10.7	8 (10.1)	3 (9.7)	27 (11.0)	
*Ethnicity*						
Dagomba/Mamprusi	273	76.7	56 (70.9)	22 (70.9)	195 (79.3)	0.007*
Frafra/Grusi	60	16.8	22 (27.8)	7 (22.6)	31 (12.6)	
Others^**††**^	23	6.5	1 (1.3)	2 (6.5)	20 (8.1)	
*Religion*						
Christianity	44	12.4	15 (19.0)	6 (19.4)	23 (9.4)	0.036*
Islamism	312	87.6	64 (81.0)	25 (80.6)	223 (90.6)	
*Employment status*						
Unemployed	142	39.9	29 (36.7)	10 (32.3)	103 (41.8)	0.354
Self-employed	181	50.8	46 (58.2)	17 (54.8)	118 (48.0)	
Public/civil servant	33	9.3	4 (5.1)	4 (12.9)	25 (10.2)	
*Household fuel*						
Firewood	148	41.6	30 (37.9)	7 (22.6)	111 (45.1)	0.004*
Charcoal	162	45.5	42 (53.2)	14 (45.2)	106 (43.1)	
Gas	46	12.9	7 (8.9)	10 (32.2)	29 (11.8)	
*Wealth quintiles*^b^						
Poorest	72	20.2	14 (17.7)	3 (9.7)	55 (22.4)	< 0.001*
Poorer	75	21.0	24 (30.4)	2 (6.4)	49 (19.9)	
Middle	70	19.7	24 (30.4)	2 (6.4)	44 (17.9)	
Richer	70	19.7	13 (16.4)	6 (19.4)	51 (20.7)	
Richest	69	19.4	4 (5.1)	18 (58.1)	47 (19.1)	
*Household size*^b^						
< 10 persons	283	79.5	71 (89.9)	25 (80.6)	187 (76.0)	0.029*
≥ 10 persons	73	20.5	8 (10.1)	6 (19.4)	59 (24.0)	
Mean = 9.83, sd = 4.79						
*Age group of neonates*						
< 7 days	106	29.8	24 (30.4)	10 (32.3)	72 (29.3)	0.935
8–28 days	250	70.2	55 (69.6)	21 (67.7)	174 (70.7)	
Mean = 6.12, sd = 2.93						
*Sex of neonates*						
Male	200	56.2	52 (65.8)	18 (58.1)	130 (52.8)	0.126
Female	156	43.8	27 (34.2)	13 (41.9)	116 (47.2)	

Birthweight (BW)	*n*	Prevalence (%)	95% CI
Low birthweight (< 2500 g)	79	22.2	17.9–26.9%
Macrosomia (≥ 4000 g)	31	8.7	4.9–12.1%
Normal BW (≥ 2500–3999 g)	246	69.1	64.0–73.8%
Mean = 2890, sd = 620			

*p value < 0.05

^**†**^Chi-square test/Fisher’s exact test

^**††**^Bulsa, Dagaati, Ewe, Fulani, Gonja

^a^Categorization based on previous studies [18, 21, 37]

### Anthropometric and antenatal characteristics of respondents

While 7.0% of the mothers were underweight, 28.7% of them were overweight. Majority of mothers (73.6%) gained at least six-kilogram body weight during pregnancy. About 53.7% of the mothers attended ANC clinics less than eight times. More than half of the mothers used insecticide-treated nets (61.2%) and ingested more than three doses of SP tablets (55.3%) during pregnancy (Table 3).

**Table 3:** Anthropometric and antenatal characteristics of respondents (*N* = 356)

Background characteristics	Frequency distribution		Bivariate analysis			
	Frequency	Proportion	Low birthweight	Macrosomia	Normal birthweight	*p* value^†^
	*n*	%	*n* (%)	*n* (%)	*n* (%)	
*Body mass index (BMI)*^a^						
Underweight (< 18.5 kg/m^2^)	25	7.0	5 (6.3)	4 (12.9)	16 (6.5)	0.448
Normal BMI (≥ 18.5 to 24.9 kg/m^2^)	229	64.3	56 (70.9)	14 (58.1)	155 (63.0)	
Overweight/Obese (≥ 25.0 kg/m^2^)	102	28.7	18 (22.8)	9 (29.0)	75 (30.5)	
Mean = 23.37, sd = 3.40						
*Pre-pregnancy weight*^a^						
< 50 kg	42	11.8	11 (13.9)	5 (16.1)	26 (10.6)	0.533
≥ 50 kg	314	88.2	68 (86.1)	26 (83.9)	220 (89.4)	
*Height at first antenatal care (ANC) visits* ^a^						
< 150 cm	13	3.7	3 (3.8)	0 (0.0)	10 (4.1)	0.522
≥ 150 cm	343	96.3	76 (96.2)	31 (100.0)	236 (95.9)	
*Gestational weight gain*^a^						
< 6 kg	94	26.4	31 (39.2)	3 (9.7)	60 (24.4)	0.003*
≥ 6 kg	262	73.6	48 (60.8)	28 (90.3)	186 (75.6)	
Mean = 6.47, sd = 2.16						
*Childbirth height/length*^b^						
≤ 47.5 cm	72	20.2	32 (40.5)	2 (6.5)	38 (15.5)	< 0.001*
> 47.5 cm	284	79.8	47 (59.5)	29 (93.5)	208 (84.5)	
Mean = 47.35, sd = 2.71						
*Number of ANC visits*						
< 8 visits	191	53.7	63 (79.7)	5 (16.1)	123 (50.0)	< 0.001*
≥ 8 visits	165	46.3	16 (20.3)	26 (83.9)	123 (50.0)	
*Place of ANC visits*						
Hospital	175	49.2	40 (50.6)	15 (48.4)	120 (48.8)	0.956
Health centre	181	50.8	39 (49.4)	16 (51.6)	126 (51.2)	
*Family planning (FP) use before last pregnancy*						
No FP use	232	65.2	50 (63.3)	20 (64.5)	162 (65.8)	0.914
FP use	124	34.8	29 (36.7)	11 (35.5)	84 (34.2)	
*Insecticide-treated nets (ITNs) use*						
No ITNs use	138	38.8	46 (58.2)	10 (32.3)	82 (33.3)	< 0.001*
ITNs use	218	61.2	33 (41.8)	21. (67.1)	164 (66.7)	
*ITNs frequency of use (n = 218)*						
Every night	179	82.1	25 (75.8)	17 (80.9)	137 (83.5)	0.562
Sometimes	39	17.9	8 (24.2)	4 (19.1)	27 (16.5)	
*Number of sulphadoxine-pyrimethamine (SP) intake*						
None	18	5.1	8 (10.1)	1 (3.2)	9 (3.7)	0.002*
1–3 doses	141	39.6	40 (50.6)	6 (19.4)	95 (38.6)	
> 3 doses	197	55.3	31 (39.2)	24 (77.4)	142 (57.7)	
*Dewormer (anthelminthics) intake*						
No intake	218	61.2	46 (58.2)	16 (51.6)	156 (63.4)	0.367
Intake	138	38.8	33 (41.8)	15 (48.4)	90 (36.6)	
*Iron/folic acid (IFA) intake*						
No IFA intake	13	3.7	4 (5.1)	0 (0.0)	9 (3.7)	0.444
IFA intake	343	96.3	75 (94.9)	31 (100.0)	237 (96.3)	
*Tetanus diphtheria (TD) immunization*						
Not immunized	26	7.3	7 (8.9)	3 (9.7)	16 (6.5)	0.679
Immunized	330	92.7	72 (91.1)	28 (90.3)	230 (93.5)	
*Reception of nutrition education*						
Not received	47	13.2	11 (13.9)	7 (22.6)	29 (11.8)	0.241
Received	309	86.8	68 (86.1)	24 (77.4)	217 (88.2)	

*p value < 0.05

^**†**^Chi-square test/Fisher’s exact test

^**a**^Categorization based on previous studies [31, 33, 38]

^**b**^Categorization based on previous studies [30]

### Obstetric and clinical characteristics of respondents

Majority of respondents were multiparous (72.7%) and multigravidae (75.3%). Preterm and post-term deliveries were 19.4% and 6.5%, respectively. Most mothers in the first (44.9%), second (56.2%) and third (44.4%) trimesters of pregnancy were anaemic. Nearly 50.0% of respondents had adequate knowledge on abnormal birthweight and its causes and effects. Few respondents had suffered from gestational diabetes (5.9%), hypertension (12.4%), hepatitis B (10.7%), and human immunodeficiency virus (1.7%) infections during pregnancy (Table 4).

**Table 4:** Obstetric and clinical characteristics of respondents (*N* = 356)

Background characteristics	Frequency distribution		Bivariate analysis			
	Frequency	Proportion	Low birthweight	Macrosomia	Normal birthweight	*p* value^†^
	*n*	%	*n* (%)	*n* (%)	*n* (%)	
*Gravidity*						
Primigravida (0–1 pregnancy)	88	24.7	18 (22.8)	4 (12.9)	66 (26.8)	0.215
Multigravida (≥ 2 pregnancies)	268	75.3	61 (77.2)	27 (87.1)	180 (73.2)	
*Parity*						
Primipara (0–1 delivery)	97	27.3	21 (26.9)	4 (12.9)	72 (29.3)	0.154
Multipara (≥ 2 deliveries)	259	72.7	58 (73.4)	27 (87.1)	174 (70.7)	
*Gestational age at birth*						
Preterm (< 37 weeks)	69	19.4	29 (36.7)	3 (9.7)	37 (15.0)	< 0.001*
Normal term (37–41 weeks)	264	74.1	48 (60.8)	18 (58.1)	198 (80.5)	
Post-term (≥ 42 weeks)	23	6.5	2 (2.5)	10 (32.3)	11 (4.5)	
*Birth order*						
First child	97	27.3	21 (26.6)	4 (12.9)	72 (29.3)	0.154
Second or more child	259	72.7	58 (73.4)	27 (87.1)	174 (70.7)	
*Knowledge level*^a^						
Inadequate	182	51.1	49 (60.0)	17 (54.8)	116 (47.1)	0.065
Adequate	174	48.9	30 (40.0)	14 (45.2)	130 (52.9)	
*First-trimester haemoglobin (Hb) levels*						
Anaemia /Low Hb (< 11 g/dL)	160	44.9	67 (84.8)	0 (0.00)	93 (37.8)	< 0.001*
Normal Hb (≥ 11–13.1 g/dL)	177	49.7	12 (15.2)	22 (71.0)	143 (58.!)	
Polycythaemia (≥ 13.2 g/dL)	19	5.4	0 (0.00)	9 (29.0)	10 (4.1)	
*Second-trimester haemoglobin levels*						
Anaemia	200	56.2	76 (96.2)	4 (12.9)	120 (48.8)	< 0.001*
Normal Hb	152	42.7	3 (3.8)	25 (80.6)	124 (50.4)	
Polycythaemia	4	1.1	0 (0.00)	2 (6.5)	2 (0.8)	
*Third-trimester haemoglobin levels*						
Anaemia	158	44.4	75 (94.9)	1 (3.2)	82 (33.3)	< 0.001*
Normal Hb	192	53.9	4 (5.1)	28 (90.3)	160 (65.0)	
Polycythaemia	6	1.7	0 (0.00)	2 (6.5)	4 (1.6)	
*Malaria episode during pregnancy*						
No episode	254	71.3	48 (60.8)	24 (77.4)	182 (74.0)	0.057
Episode	102	28.7	31 (39.2)	7 (22.6)	64 (26.0)	
*Gestational diabetes*						
Without diabetes	335	94.1	79 (100.0)	26 (83.9)	230 (93.5)	0.002*
With diabetes	21	5.9	0 (0.00)	5 (16.1)	16 (6.5)	
*Hypertension (HPT) status*						
Without HPT	312	87.6	68 (86.1)	22 (72.0)	222 (90.2)	0.008*
With HPT	44	12.4	11 (13.9)	9 (29.0)	24 (9.8)	
*Syphilis infection (n = 345)*						
Without syphilis	342	99.1	78 (98.7)	30 (96.8)	234 (99.6)	0.262
With syphilis	3	0.9	1 (1.3)	1 (3.2)	1 (0.4)	
*HIV infection*						
Without HIV	350	98.3	79 (100.0)	31 (100.0)	240 (97.6)	0.256
With HIV	6	1.7	0 (0.00)	0.(0.00)	6 (2.4)	
*Hepatitis B infection*						
Without hepatitis B	318	89.3	67 (84.8)	28 (90.3)	223 (90.6)	0.337
With hepatitis B	38	10.7	12 (15.2)	3 (9.7)	23 (9.4)	
*Sickle cell status*						
Without sickle cell	301	84.5	65 (82.3)	28 (90.3)	208 (84.5)	0.576
With sickle cell	55	15.5	14 (17.7)	3 (9.7)	38 (15.5)	
*Blood rhesus type (n = 343)*						
Rhesus negative	53	15.5	13 (16.5)	1 (3.2)	39 (16.7)	0.142
Rhesus positive	290	84.5	66 (83.5)	30 (96.8)	194 (83.3)	
*G6PD status*						
Normal	333	93.5	71 (89.9)	30 (96.8)	232 (94.3)	0.282
Complete/partial	23	6.5	8 (10.1)	1 (3.2)	14 (5.7)	

*p value < 0.05

^**†**^Chi-square test/Fisher’s exact test

^**a**^Categorization based on previous studies [36]

### Maternal risk factors for low birthweight and macrosomia

Table 5 shows the results of analyses of significant maternal risk factors for low birthweight and macrosomia. Of the total background (independent) variables examined on bivariate association with abnormal birthweight, 16 variables were significantly associated (*p* < 0.05) as shown in Tables 2, 3, and 4. Due to multicollinearity, one variable (thus, second-trimester haemoglobin levels) was excluded from the significant independent variables for the multinomial logistic regression model.

**Table 5:** Analysis of maternal risk factors for low birthweight and macrosomia (*N* = 356)

Characteristics	Multinomial logistic regression (Normal birthweight = base outcome)							
	Low birthweight				Macrosomia			
	COR	*p* value (95% CI )	AOR	*p* value (95% CI )	COR	*p* value (95% CI )	AOR	*p* value (95% CI )
*Ethnicity*								
Dagomba				[Reference]				[Reference]
Frafra/Grusi	2.471	0.091 (1.327–4.603)	1.231	0.679 (0.460–3.298)	2.001	0.144 (0.789–5.078)	1.207	0.811 (0.258–5.642)
Others^**††**^	0.174	0.004 (0.023–1.326)*	**0.104**	**0.039 (0.011**–0.995)*	0.886	0.876 (0.194–4.048)	0.795	0.821 (0.109–5.817)
*Religion*								
Christianity				[Reference]				[Reference]
Islamism	0.440	0.023 (0.217–0.893)*	0.389	0.119 (0.118–1.277)	0.430	0.094 (0.160–1.155)	0.471	0.348 (0.098–2.265)
*Household fuel*								
Firewood				[Reference]				[Reference]
Charcoal	1.466	0.164 (0.855–2.513)	0.891	0.802 (0.360–2.201)	2.094	0.125 (0.814–5.391)	0.801	0.766 (0.187–3.431)
Gas	0.893	0.809 (0.356–2.238)	1.470	0.670 (0.251–8.621)	5.468	0.001 (1.916–15.61)*	2.058	0.413 (0.366–11.58)
*Wealth quintile*								
Poorest	0.467	0.052 (0.216–1.007)	0.717	0.591 (0.214–2.406)	1.200	0.845 (0.192–7.500)	2.352	0.480 (0.219–25.21)
Poorer	0.898	0.762 (0.447–1.803)	0.702	0.519 (0.239–2.060)	0.898	0.916 (0.121–6.647)	0.862	0.913 (0.059–12.38)
Middle				[Reference]				[Reference]
Richer	0.467	0.058 (0.213–1.026)	0.629	0.439 (0.195–2.034)	2.588	0.259 (0.497–13.48)	2.579	0.380 (0.311–21.41)
Richest	0.156	0.001 (0.050–0.486)*	0.295	0.175 (0.050–1.725)	8.426	0.006 (1.847–38.43)*	**14.25**	**0.016 (1.638**–23.91)*
*Household size*								
< 10 persons				[Reference]				[Reference]
≥ 10 persons	0.357	0.010 (0.163–0.785)*	0.681	0.517 (0. 212–2.181)	0.761	0.568 (0.298–1.943)	0.804	0.752 (0.208–3.110)
*Weight gain*								
< 6 kg				[Reference]				[Reference]
≥ 6 kg	0.499	0.011 (0.292–0.855)*	0.639	0.292 (0.279–1.468)	3.011	0.078 (0.884–10.26)	0.1667	0.066 (0.247–1.124)
*Childbirth length*								
≤ 47.5 cm				[Reference]				[Reference]
> 47.5 cm	0.268	< 0.001 (0.152–0.473)*	**0.271**	**0.003 (0.113**–0.651)*	2.649	0.195 (0.607–11.57)	0.956	0.961 (0.158–5.782)
*Number of ANC visits*								
< 8 visits				[Reference]				[Reference]
≥ 8 visits	0.254	< 0.001 (0.139–0.464)*	**0.249**	**0.002 (0.103**–0.602)*	5.200	0.001 (1.934–13.98)*	0.804	0.752 (0.208–3.110)
*ITNs use*								
ITNs use				[Reference]				[Reference]
No ITNs use	2.788	< 0.001 (1.658–4.688)*	0.931	0.863 (0.413–2.097)	0.952	0.905 (0.429–2.116)	0.970	0.962 (0.275–3.423)
*Number of SP intake*								
None	2.111	0.152 (0.760–5.863)	4.066	0.157 (0.584–28.30)	1.759	0.619 (0.190–16.27)	0.765	0.862 (0.037–15.63)
1–3 doses				[Reference]				[Reference]
> 3 doses	0.518	0.016 (0.303–0.886)*	1.470	0.368 (0.635–3.403)	2.676	0.038 (1.054–6.793)*	0.745	0.674 (0.190–2.926)
*Gestational age at birth*								
Preterm	3.233	< 0.001 (1.811–5.771)*	1.548	0.348 (0.621–3.858)	0.892	0.860 (0.250–3.181)	4.980	0.071 (0.873–28.41)
Normal term				[Reference]				[Reference]
Post-term	0.750	0.714 (0.161–3.495)	2.709	0.502 (0.148–49.71)	10.00	< 0.001 (3.742–26.72)*	**23.21**	** < 0.001 (4.603**–56.19)*
*First-trimester Hb levels*								
Anaemia	8.585	< 0.001 (4.404–16.74)*	**3.226**	**0.007 (1.372**–7.584)*	–	0.974 (–)	–	0.988 (–)
Normal Hb				[Reference]				[Reference]
Polycythaemia	–	0.997 (–)	–	1.000 (–)	5.852	0.001 (2.139–16.00)*	3.460	0.128 (0.700–17.09)
*Second-trimester Hb levels*								
Anaemia	26.17	< 0.001 (8.038–85.22)*	Dropped due to multicollinearity		0.165	0.001 (0.559–0.489)*	Dropped due to multicollinearity	
Normal Hb		[Reference]				[Reference]		
Polycythaemia	–	0.986 (–)			4.964	0.118 (0.667–36.92)		
*Third-trimester Hb levels*								
Anaemia	36.57	< 0.001 (12.92–103.5)*	**23.94**	** < 0.001 (7.442**–77.01)*	0.070	0.009 (0.009–0.521)	0.116	0.074 (0.011–1.230)
Normal Hb				[Reference]				[Reference]
Polycythaemia	–	0.993 (–)	–	0.999 (–)	2.859	0.238 (0.500–16.36)	0.485	0.559 (0.043–5.500)
*Gestational diabetes*								
Without diabetes				[Reference]				[Reference]
With diabetes	–	0.982 (–)	–	0.996 (–)	2.767	0.035 (0.937–8.169)*	1.058	0.941 (0.236–4.738)
*Hypertension status*								
Without HPT				[Reference]				[Reference]
With HPT	1.496	0.301 (0.697–3.211)	2.787	0.161 (0.665–11.67)	3.784	0.003 (1.566–9.146)*	2.581	0.158 (0.692–9.632)
*Regression model*								
*R*^2^	0.477							
*p* value	< 0.001							

Bold = significant AOR, p value, and 95%CI for risk factors

AOR, Adjusted odds ratio; COR, Crude odds ratio

*p value < 0.05

^**††**^Bulsa, Dagaati, Ewe, Fulani, Gonja

The model registered significant adjusted odds ratio (AOR) (*p* < 0.05) for independent factors including other (minor) ethnic groups, ANC visits ≥ 8, childbirth length > 47.5cm, and maternal anaemia in first and third trimesters of pregnancy for low birthweight (Table 5). The result suggests that mothers from minor ethnic groups located in Savelugu municipality were 89.6% [= (1–0.104) × 100] less likely to deliver low birthweight newborns (95% CI 0.011–0.995). Mothers who attended eight ANC visits or more reduced the risk of giving birth to low birthweight newborns by 75.1% [= (1–0.249) × 100] (95% CI 0.103–0.602). Moreover, mothers with anaemia in the first and third trimesters of pregnancy were 3.226 (95% CI 1.372–7.584) and 23.94 (95% CI 7.442–77.01) times more likely to deliver low birthweight newborns, respectively. It was also discovered that mothers having neonates whose birth length was greater than 47.5cm were less likely to deliver low birthweight newborns, indicating 72.9% [= (1–0.271) × 100] reduction in delivering low-weight newborns (95% CI 0.113–0.651).

Additionally, the model identified post-term delivery and richest wealth quintile (highest socioeconomic status) as the significant risk factors for macrosomia (*p* < 0.05) (Table 5). The analysis reveals that mothers with advanced gestational age (≥ 42 weeks) were 23.21 times more likely to deliver macrosomic children (95% CI 4.603–56.19). Mothers from the richest households (highest socioeconomic homes) were more likely to give birth to macrosomic children (AOR: 14.25; 95% CI 1.638–23.91).

## Discussion

The prevalence rates of low birthweight and macrosomia were 22.2% and 8.7%, respectively. This places the study municipality at higher risk for perinatal mortality and adult chronic diseases in future [3, 6]. The prevalence of low birthweight in this study was found in the range (12–24%) reported in some developing countries [39, 40]. The diverse diet practices and education status of the studied mothers coupled with different health service delivery systems across countries could be responsible for this variation. Despite possible overestimation of our findings owing to seasonality, the prevalence of low birthweight in this study was also greater than the national prevalence (10.1%) [23] and that in the Hohoe (9.7%) [12] and Dodowa (7.52%) [41] municipalities (9.7%) of Southern Ghana. This might be attributable to poor socioeconomic status of mothers in Savelugu municipality [23] as poverty has frequently been correlated with increased odds of low birthweight [33]. Additionally, unhealthy food practices like pica (thus, perversion of appetite for ice, clay, soap, chalk) is common among pregnant mothers in Savelugu municipality [42] which could be responsible for the higher low birthweight prevalence [43]. Inconsistent with some findings in Nigeria [10] and China [11], macrosomia prevalence was higher in our study. The present study used only singleton live births which is likely to increase macrosomia prevalence as compared to the previous studies [10, 11]. The prevalence of macrosomia in this study was lower than that in Kumasi city (11%) of  Southern Ghana [44]. Pre-pregnancy overweight/obesity which has been reported as a risk factor for macrosomia [10] is more prevalent in Kumasi (52.6%) [44] than the prevalence (28.7%) found in our study, though our study showed no significant association between overweight/obesity and macrosomia.

In this study, the major ethnic group found was Dagomba/Mamprusi (273; 76.7%), while Frafra/Grusi (60; 16.8%) was the second largest group. Our study indicated that mothers belonging to other (minor) ethnic groups (Bulsa, Dagaati, Ewe, Fulani, and Gonja) were less likely to give birth to low-weight children, which is dissimilar to previous studies [45, 46]. This could be accountable to not only the unhealthy diet practices among the major ethnic groups but also the differences in the socio-cultural and health practices among the various ethnic groups in the municipality. Moreover, of the 23 mothers from minority ethnic groups, the proportion of mothers who had formal education (20; 87.0%) was greater than those without formal education (*p* = 0.028 in *χ*^2^/Fisher’s exact test). There was also significant association between socioeconomic status (wealth quintile) and minor ethnic groups (*p* = 0.047 in *χ*^2^/Fisher’s exact test). Thus, education and socioeconomic status of mothers from minor ethnic groups tend to have protection against malnutrition [32, 47], thereby leading to the prevention of low birthweight.

WHO proposed the “2016 WHO ANC model” describing a new series of recommendations to improve the quality of ANC for progressive pregnancy outcomes [34]. This model recommends a minimum of eight ANC visits as Ghana has adopted it as the national standard [48]. Meanwhile, most countries are still obliged to the former model of four visit-focused ANC (FANC). Our study identified that mothers who made eight ANC visits or more had a reduced risk of giving birth to low-weight children. A multicentre study in Africa revealed that mothers who had four ANC visits or more had reduced risks of abnormal birth outcomes [49]. Less than half of mothers (165; 46.3%) in this study made eight ANC visits or more. This relatively low proportion (46.3%) is a greater concern that calls for the need to promote the importance of ANC through community education and constantly assess the factors that limit ANC attendance. This is because a decrease in frequency of ANC visits reduces the contents of care at health facilities and also increases the risk of low birthweight [50]. Additionally, ANC visits create greater opportunities for screening potential risk factors in pregnancy and offering some preventive interventions to avoid small-weight children [34]. These risk factors may remain undetected if pregnant mothers do not make ANC visits or had lesser visits [34, 50]. However, frequency of ANC visits is not just enough for preventing low birthweight but placing more emphasis on the quality of ANC is paramount, especially in developing countries.

The study revealed that mothers having children whose birth length was greater than 47.5cm were less likely to deliver low-weight children. This finding is in contrast to a study in Brazil [30]. The length of a child at birth needs to be supported by the mother’s nutritional and environmental practices during pregnancy, which is determined in utero through foetal programming [3, 9]. Thus, mothers expecting taller newborns (childbirth length > 47.5cm) could be accompanied by appropriate maternal nutritional and other environmental practices during gestation. Mothers’ height has been reported as a genetic proxy for predicting children’s height [51]. The mean height of the mothers in our study was 160.97cm (sd = 6.25cm), and statistical analysis showed that there was significant correlation between maternal height and childbirth length (*r*_*s*_ = 0.175; *p* = 0.001). Therefore, mothers are slightly likely to possess greater height to genetically deliver tall children which serves as protection against low birthweight. However, length measurements of children at health facilities in the municipality may be over/underestimated due to possible errors and inaccuracies from the health workers. It usually becomes difficult for making children fully stretched during length measurement [52].

Globally, anaemia during pregnancy is considered a public health concern, especially in developing countries [53]. In our study, mothers with haemoglobin levels less than 11 g/dL in the first trimester of gestation were more likely to deliver low birthweight newborns. Some meta-analyses and systematic reviews [54, 55] are confirmatory to our study, while prospective study in four developed countries was inconsistent [56]. Though anaemia is a strong predictor of maternal undernutrition, iron deficiency anaemia is purportedly the commonest cause in pregnancy [57]. Anaemia directly causes poor foetal development due to insufficient oxygen supply to the placental tissues [58] which causes low birthweight [3, 9]. In this study, of the 160 mothers who had gestational anaemia in their first trimester, greater proportion (107; 66.9%) did not ingest deworming tablets (anthelmintic drug) during pregnancy as few mothers took the drug (*p* = 0.048 in *χ*^2^/Fisher’s exact test). Additionally, 111 (69.4%) of these first-trimester anaemic mothers made less than eight ANC visits, while the rest made eight ANC visits or more (*p* = 0.001 in *χ*^2^/Fisher’s exact test). These earlier studies [49, 50, 59] support our study. Thus, non-intake of anthelmintic drugs and lesser ANC visits tend to increase the risk of gestational anaemia among the mothers, leading to the birth of low-weight children.

Furthermore, mothers with anaemia in the third trimester of pregnancy had higher risk of giving birth to small-weight children. Not only were the results similar to a systematic review by Sukrat et al. [60] but also inconsistent with retrospective report in Pakistan [61]. During gestation, there is a physiological fall in haemoglobin levels from the first to the third trimester usually estimated at 5–14 g/dL [58]. This is attributable to the rise in plasma volume surpassing the increase in red cell mass [62] leading to birth of abnormal birthweight children [9]. Of the 158 anaemic mothers found in the third trimester of pregnancy, the proportion of those who did not use insecticide-treated bed nets (ITNs) (81; 51.3%) is greater than those who used it (*p* = 0.001 in *χ*^2^/Fisher’s exact test). Larger segment (102; 64.6%) of these third-trimester anaemic mothers made less than eight ANC visits, while few made eight ANC visits or more (*p* = 0.001 in *χ*^2^/Fisher’s exact test). Lesser ANC visits and non-use of ITNs by mothers during pregnancy increased the risk of developing anaemia [23, 49, 50] which upsurges low birthweight prevalence.

Alternatively, the present study indicated that mothers with gestational age equal to or more than 42 weeks had higher risk of giving birth to macrosomic children. Giving birth at late gestational age is associated with maternal and perinatal deaths [63]. Post-term gestation increases macrosomic risk which has been confirmed in meta-analysis in Africa and Asia [10, 64]. An advanced gestational age could lead to the birth of large-weight children through continual promotion of the uterine growth process. This is to be expected as newborns experience weight gain between 150 g and 200 g at term [65] or sometimes around 115–242 g [66]. Hence, advanced gestational age increases the probability for extra weight gain as foetus continuously stays in the uterus.

Mothers from the highest socioeconomic homes (richest households) were more likely to give birth to macrosomic children. This finding is in conformity with some community-based studies in India [67] and Canada [68] but in contrast to a study in  Southern Ethiopia and Ghana [37, 41]. Though mothers from the highest socioeconomic class have been established to influence macrosomic births in most developing countries [20, 67], this study used household socioeconomic characteristics as a proxy to determine the socioeconomic status of the mothers. Hence, further studies are recommended to investigate this covariate among the studied mothers in the municipality.

Our study encountered some limitations. This study used existing anthropometric data (weight, height/length, birthweight) recorded in the MCHRB. Some of these data might not be correct due to mis-transcription, mis-recording, and mismeasurement by non-standardized methods. The study results could be under/overestimated due to the time/season of data collection. The data were collected from February to March 2022, the period of dry season in Savelugu municipality, Ghana. Since that period is a non-farming season, there is high cost of food due to scarcity of food commodities. This prevents most pregnant and nursing mothers from having access to diversified meals. Hence, this could lead to malnutrition among the mothers that could affect birth outcomes through foetal programming. Moreover, during this season, most pregnant and nursing mothers are prone to mild infections such as respiratory, malaria, diarrhoea, and other systemic infections which affect their nutritional status leading to abnormal birthweight. Meanwhile, data on maternal nutritional status and seasonal infections were not prospectively collected to determine possible correlations.

Secondly, possible sampling bias could occur due to non-selection of mothers having lost or misplaced their MCHRB, absented from first-trimester ANC and PNC services, and with home deliveries. Finally, the study has limitations in generalizability due to the employment of cross section as the study design. Hence, it would need regional and/or national surveys to obtain generalizable findings and conclusions.

## Conclusion

The study demonstrated the prevalence of abnormal birthweight and the associated risk factors for abnormal birthweight among lactating mothers having a newborn in the past four weeks. The prevalence rates of low birthweight and macrosomia were relatively high. The present study identified that mothers from minority ethnic groups belonging to Bulsa, Dagaati, Ewe, Fulani, and Gonja; mothers who made eight ANC visits or more; and mothers having children born with length above 47.5 cm were less likely to deliver low birthweight children. Maternal anaemia in the first and third trimesters of pregnancy increased the risk of delivering low-weight children. Alternatively, mothers with advanced gestational age and those from richest households had higher risk of giving birth to macrosomic children.

Our findings provide information for the Ghana Ministry of Health (MOH) particularly through Ghana Health Service (GHS) to be strongly committed to training and employing more public health nutritionists (PHNs) in its health service delivery system. Specifically, PHNs would educate and counsel semi-rural communities and pregnant women on appropriate diet practices in addressing most nutrition-related determinants of abnormal birthweight including anaemia. Moreover, enhancing the capacity of community health nurses and midwives should be considered to provide appropriate nutrition counselling to pregnant mothers in the health facilities. The MOH should enforce existing policies to strengthen obstetric protocols in the health system. Organizing regular community engagement on education and promotion of ANC visits in semi-rural communities should be collaborative efforts between GHS and local governments.

It also becomes incumbent on the Ministry of Education (through Ghana Education Service) to introduce nutrition courses in the education curriculum at elementary, junior, and senior high schools. Children who are our future adults will be equipped with practical diet habits and nutrition knowledge on Developmental Origin of Health and Diseases (DOHaD) to improve the life course approach through the prevention of abnormal birthweight.

## Data Availability

The datasets collected, generated, and/or analysed during the present study are only available from the corresponding author upon reasonable request.

## Abbreviations

ANC: Antenatal care
BMI: Body mass index
FANC: Focused antenatal care
GDM: Gestational diabetes mellitus
GHS: Ghana health service
GWG: Gestational weight gain
LBW: Low birthweight
ITNs: Insecticide-treated bed nets
MCHRB: Maternal and child health record book
MCS: Macrosomia
MOH: Ministry of health
ODK: Open data kit
PHNs: Public health nutritionists
PNC: Postnatal care
STATA: Statistics and data
WHO: World health organization
